# Effectiveness of Ternary Blend Incorporating Rice Husk Ash, Silica Fume, and Cement in Preparing ASR Resilient Concrete

**DOI:** 10.3390/ma15062125

**Published:** 2022-03-14

**Authors:** Ali Ahmed, Shoaib Ameer, Safeer Abbas, Wasim Abbass, Afia Razzaq, Abdeliazim Mustafa Mohamed, Abdullah Mohamed

**Affiliations:** 1 Department of Civil Engineering, University of Engineering and Technology, Lahore 54890, Pakistan; ali@uet.edu.pk (A.A.); safeerabbas26@gmail.com (S.A.); 2 Department of Civil Engineering, National University of Computer and Emerging Sciences (FAST-NU), Lahore 54770, Pakistan; shoaibameeruet@gmail.com; 3 Department of Architectural Engineering & Design, University of Engineering and Technology, Lahore 54890, Pakistan; afiarazzaq@uet.edu.pk; 4 Department of Civil Engineering, College of Engineering in AlKharj, Prince Sattam Bin Abdulaziz University, Alkharj 16273, Saudi Arabia; a.bilal@psau.edu.sa; 5 Building & Construction Technology Department, Bayan University, Khartoum 210, Sudan; 6 Research Centre, Future University in Egypt, New Cairo 11745, Egypt; mohamed.a@fue.edu.eg

**Keywords:** rice hush ash, silica fume, ternary blend, reactive aggregate, concrete, durability, alkali-silica reaction, expansion, sustainability

## Abstract

Although the disposal of waste ashes causes environmental hazards, recycling them helps in reducing their harmful impacts and improves the characteristics of building materials. The present study explores the possible use of locally available waste ashes including Rice husk ash (RHA)and Silica Fumes (SF) as a partial replacement for cement in concrete to counter the negative impact of alkali-silica reactions (ASRs). In the present study, ternary blends including RHA (0–30%), SF (5% and 10%) and Portland cement were investigated. The amorphous behavior of RHA and SF was confirmed by conducting an X-ray diffraction analysis. A petrography analysis was carried out to ensure the reactive nature of aggregates used to prepare the concrete specimen. Accelerated mortar bar tests were performed in accordance with ASTM C 1260 for up to 90 days. It was revealed that specimens incorporating a ternary blend of SF, RHA, and Portland cement exhibited less expansion compared to the control specimens without SF and RHA. The incorporation of 5% SF along with 20% RHA exhibited a 0.13% expansion at 28 days and 10% SF, along with 5% RHA which exhibited 0.18% expansion at 28 days which is within the range specified by ASTM C 1260, with the lowest compromise of the mechanical properties of concrete. Thus, the utilization of SF and RHA in the partial replacement of cement in concrete may be considered a practical approach to mitigate ASR effects as well as to reduce the environmental burden.

## 1. Introduction

Alkali silica reaction (ASR), commonly termed concrete cancer, is a major challenge faced by engineers globally. ASR in concrete structures may lead to cracking in concrete, spalling, as well as serviceability issues. Furthermore, ASR may directly support the corrosion of steel inside reinforced concrete members and cause further damage. The key components involved in the ASR reaction are alkalis present in the pore solution and reactive aggregates. ASR gel is the product of this reaction which tends to expand in the presence of water. As a result, the expansion causes the surrounding concrete to crack, giving rise to secondary problems including the ingress of harmful environmental elements which may support the steel rebar corrosion [1,2]. Previous studies have reported a tremendous reduction in the compressive and flexural properties of concrete due to ASR. Safeer et al. reported a reduction of up to 16% in the compressive strength of concrete due to ASR [3]. A similar reduction in mechanical properties was reported by several researchers [1,2,4].

It has been reported that ASR has affected many structures globally. These include many bridges in Japan, pavements in the US and Canada, bridges in Poland and Norway, etc. [5,6,7,8,9]. Similarly, various structures in Pakistan were also affected by ASR, i.e., Tarbela Dam, Mangla Dam, and Warsak Dam [10]. Therefore, there is a need to explore the incorporation of admixtures to control the harmful effects of ASR on concrete.

Due to the rise of the industrial revolution, the quantity of waste ashes being produced has increased tremendously. Furthermore, the dumping of these ashes in open landfills gives rise to environmental and health-related issues. Therefore, the recycling of such ashes in industrial products is considered urgent. Various researchers have reported the benefits of utilizing waste materials in concrete. For instance, Ferdous et al. reported that ceramic powder can be utilized in concrete to enhance the bending modulus. Furthermore, a good balance of the physical and mechanical properties could be achieved [11]. Similarly, a detailed review on the use of waste glass in concrete was provided by Siddika et al. [12]. Numerous supplementary cementitious materials (SCMs) have been used in concrete to control the ASR including metakaolin, fly ash, silica fume, and slag produced from blast furnaces [12,13,14,15,16]. However, the use of ternary blends that incorporate cement and waste products requires further exploration. In this study, locally available silica fume (SF) and rice husk ash (RHA) have been explored as partial replacement materials in cement in concrete.

SF can be considered a byproduct owing to its pozzolanic nature. It has amorphous properties and is obtained as a byproduct during the manufacturing of various silicon and ferrosilicon compounds. SF is considered one of the most efficient supplementary cementitious materials due to its ability to enhance the mechanical and durability properties of concrete. Silica fume has been reported to effectively mitigate expansion in concrete due to ASR [17,18,19]. Moreover, the annual generation of rice is around 600 million tons across the world. Rice processing produces husk as a byproduct which is used as a source of heat in several industries. In RHA, there is a 75–80% silica content if the temperature is no more than 700 °C. As RHA silica mostly shows 85–90% amorphous behavior, it is reactive under alkaline conditions [20]. By using fine RHA as a cementitious material, the porosity of the concrete structure is reduced, and a denser material is produced, which enhances the durability and mechanical-strength properties of the concrete.

Both Silica Fume (SF) and Rice husk ash (RHA) have been used to mitigate ASR expansion. Substantial work has been carried out to mitigate ASR from concrete using various techniques, including the usage of binary blends. However, limited research is available regarding the use of ternary blends in attenuating the effects of ASR. In this research, a ternary blend of RHA, SF, and cement is used to mitigate ASR and to enhance the mechanical properties (Compressive and Flexural strength). This study is primarily aimed at the exploration of the effectiveness of ternary blends including waste materials and ordinary Portland cement in controlling the deleterious effects of ASR in concrete. It is believed that this research will assist construction engineers and stakeholders when utilizing waste RHA and SF in order to attenuate the effects of ASR at a lower cost and to reduce the environmental pollution causes by RHA deposition in open landfill areas.

## 2. Materials and Mixture Proportions

### 2.1. Materials

**Figure 1:**
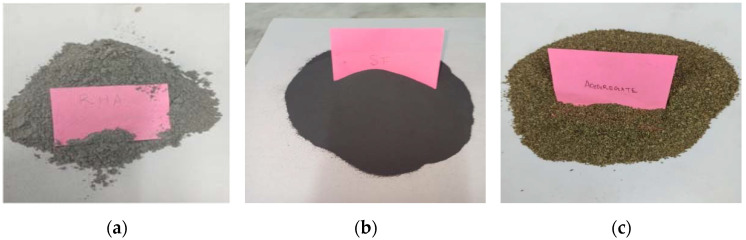
Physical appearance of raw material.(**a**) Rice Husk Ash; (**b**) Silica Fumes; (**c**) Aggregates.

Locally available reactive aggregates from Chak-110, Sargodha, Pakistan were procured to ensure ASR in the control specimen. The aggregates were later dried in a laboratory oven and crushed and sieved to achieve the aggregate gradation as prescribed by ASTM C1260 [21]. Rice husk ash (RHA) and Silica fume (SF) were collected from the local industry. These materials were selected based on the previous literature indicating the role of these waste materials in controlling ASR in concrete. Afterward, rice husk ash was further processed to reduce the particle size to a range between 10 µm and 75 µm. Figure 1 shows the aggregates, RHA, and SF used. Commercially available ordinary Portland cement was used in the concrete mixtures. RHA was used to replace 0–30% of cement in various mixtures, while SF was used to replace 5% or 10% of cement in addition to RHA replacement. The detailed mixture proportions are presented in Table 1. Concrete mixtures were prepared following the specifications mentioned in ASTM C 1260, such as a water-to-cement ratio (w/c) of 0.47 and cement-to-aggregate ratio of 1:2.25.

### 2.2. Tests on Raw Materials

The composition of pozzolanic materials (Portland Cement, SF, and RHA) was determined using the X-ray diffraction technique (XRD). Furthermore, the soundness of cement was obtained using the procedure described by ASTM C151 [22]. The relative density and fineness of raw materials were examined following ASTM C188 [23] and ASTM C184 [24], respectively. The mineralogical composition of aggregates used in concrete mixtures was determined by performing a Petrographic examination in accordance with ASTM C295 [25], while the specific gravity and water absorption of aggregates were examined in accordance with ASTM C127 [26]. The percentage of voids present in aggregates and bulk density were evaluated by following ASTM C29 [27]. Abrasion strength, crushing strength, and toughness of aggregates were determined through ASTM C535 [28], BS-812-110 [29], and BS-812-112 [30], respectively.

### 2.3. Fresh Properties of Mixtures Incorporating RHA and SF

The various tests were performed following the ASTM standard procedure to explore the performance of fresh mortar for different proportions of SF, RHA, and potentially reactive aggregates. Tests for normal consistency and setting time were performed in accordance with ASTM C187 [31] and ASTM C191 [32], respectively. The workability of various mortar mixtures incorporating SF and RHA was evaluated by following ASTM C1437 [33]. Furthermore, flowability was measured for a fixed water-cement ratio (w/c) of 0.47 (as mentioned in ASTM C1260 [34]).

### 2.4. Cube and Prism Specimens

Five cube specimens (50 × 50 × 50 mm) and five prismatic specimens (40 × 40 × 160 mm) were prepared corresponding to each testing age (7, 14, 28, 56, and 90 days) and each of the mixtures, as shown in Table 1. Casting was carried out following two layers of concrete filling along with compaction carried out utilizing a vibrating table. The specimens were cured by submersion in a water tank until the testing age. A compressive strength test was performed on mortar cubes following ASTM C109 [35], while the Modulus of rupture test was performed on mortar prisms following ASTM C348 [36]. Furthermore, a thermo-gravimetric analysis (TGA) and differential thermal analysis (DTA) were performed to investigate pozzolanic behavior. Mass loss and change in mineralogical composition were examined for specimens incorporating 5% SF along with 20% RHA, 10% SF along with 5% RHA and for the control specimen at 1200 °C.

### 2.5. Mortar Bar Specimens

**Figure 2:**
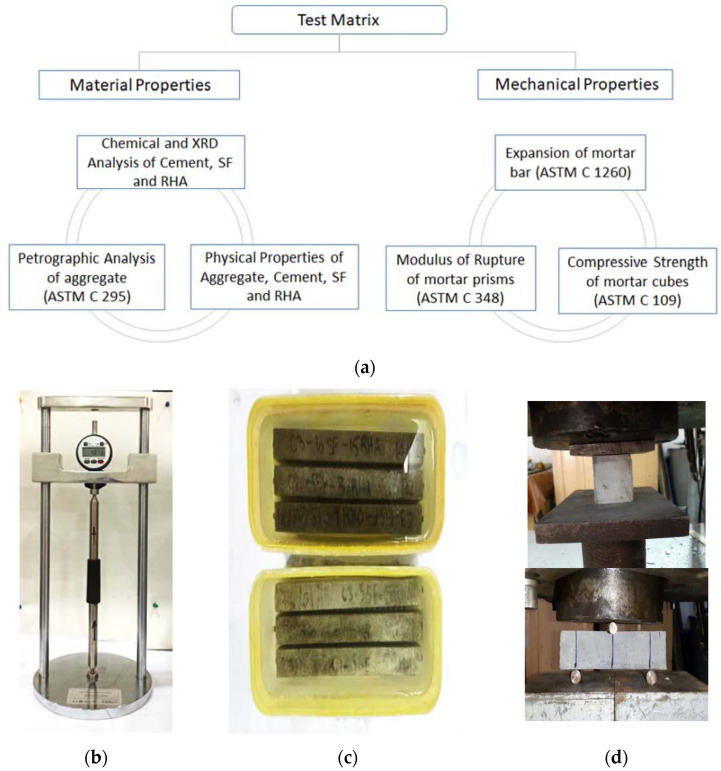
Tested Specimen (**a**) Flow chart of test matrix (**b**) Length Comparator (**c**) Mortar Bar Specimen (**d**) Cube and MOR specimen.

Mortar bar specimens (25 × 25 × 285 mm) were cast to determine the expansion caused by the alkali-Silica Reaction (ASR) in accordance with ASTM C1260 [34]. Mixing was performed by following ASTM C305 [37]. Five mortar bars were prepared for each combination, as detailed in Table 1. A length comparator was used to measure the length change in the mortar bar in accordance with ASTM C490 [38]. Mortar bar specimens were placed in an air-tight container with a solution of 1N NaOH concentration at 80 °C for a duration of 28 days. Length change of mortar bars was examined after 3, 7, 14, 21, and 28 days. Figure 2 shows the various specimen tested and the length comparator used to determine the expansion in mortar bar specimen.

## 3. Results and Discussion

### 3.1. Material Characterization

Table 2 shows the chemical composition of the used cement, SF, and RHA. The combined sum of SiO_2_, Al_2_O_3,_ and Fe_2_O_3_ was 89 for RHA and 91 for SF, which indicated that both SF and RHA may be categorized as pozzolanic materials as per ASTM C 618 [39]. The LOI of both SF and RHA was less than 10%. Moreover, chemical analysis results showed that all components were present within the range specified by ASTM C114 except alkalis [40], and comparable to the previous findings [41,42]. For instance, Abbas et al. reported a silica content of 21.1% and 76.8% in cement and RHA, respectively, which is similar to the findings reported in Table 2 of this study. Similarly, Abbas et al. [41]. reported a CaO content of 61.7% and 3.25% in cement and RHA, respectively, which is similar to the findings of reported in Table 2 which are 59.6% and 0.65% [41]. Furthermore, Bidare et al. reported silica content ranging from 90 to 96% in silica fumes and LOI content ranging from 0.1 to 0.6%. These values are consistent with the results reported in the current study.

**Figure 3:**
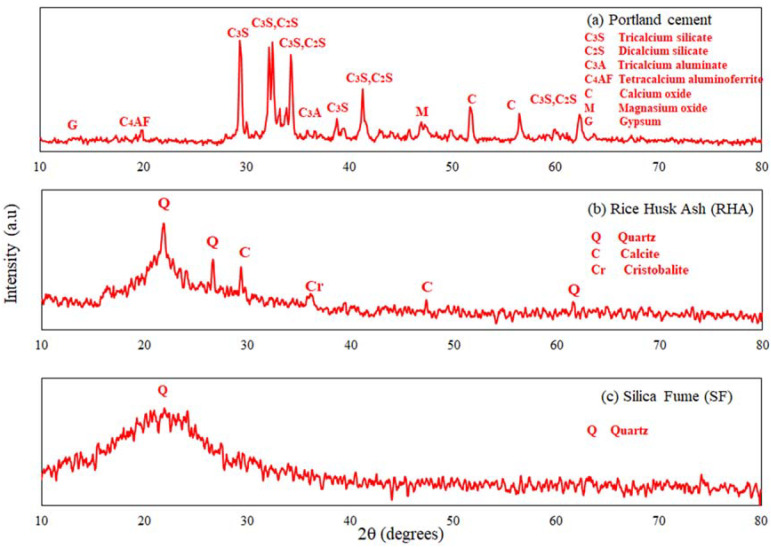
XRD Analysis of (**a**) Cement (**b**) Rice Husk Ash and (**c**) Silica Fume.

Figure 3 shows the X-ray diffraction (XRD) results of cement, RHA, and SF. It was identified that OPC consisted of tri-calcium silicate (C_3_S), di-calcium silicate (C_2_S), tri-calcium aluminate (C_3_A), tetra-calcium alumino-ferrite (C_4_AF), oxides of calcium and magnesium along with traces of gypsum. However, the pattern of RHA indicated the existence of quartz along with calcite and cristobalite, which was in agreement with previous studies [43,44]. Furthermore, SF showed amorphous behavior due to the presence of quartz [45].

Table 3 shows the physical properties of cement, SF, and RHA. The specific gravity value of cement (3.12) was higher than SF (2.25) and RHA (2.09). Moreover, the unit weight results of SF and RHA (580.4 kg/m^3^ and 554.8 kg/m^3^) were less than the cement (1428 kg/m^3^). Therefore, the utilization of SF and RHA can produce lighter structures. The autoclave expansion of cement was found to be 0.11%, which was within limits (less than 0.2%) in accordance with ASTM C1260 [34]. Therefore, the cement used during this research work was appropriate to identify the alkali-silica reaction.

Table 4 shows the results of the physical properties of the aggregate sample. All the physical properties were within specified limits in accordance with ASTM specifications. For instance, water absorption was noticed 0.96%, i.e., lesser than ASTM C33 (1%) [46]. Aggregate bulk density was noticed within the ASTM limit (i.e., 1393 kg/m^3^). Furthermore, the impact and crushing values of aggregates were 22% and 26%, respectively.

**Figure 4:**
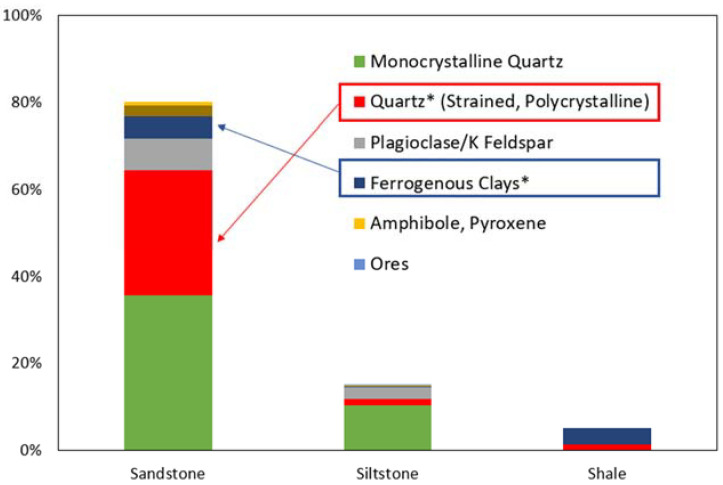
Petrographic Analysis of Aggregate (ASTM C 295) (* indicates the reactive components).

Petrographic examination results have been shown in Figure 4. It was identified that the aggregates consisted of three major types of rocks, i.e., sandstone (80%), siltstone (15%), and shale (5%). Furthermore, the aggregates were found to consist of polycrystalline and strained quartz. Polycrystalline quartz was around 35% of the sub-group sandstone and 9% of the sub-group siltstone. The existence of such quantities of reactive quartz in aggregates indicates that the used aggregates have the potential for developing ASR in concrete.

### 3.2. Fresh Properties of Mixtures Incorporating SF and RHA

Table 5 shows the flow of mixtures incorporating various proportions of SF and RHA. It was observed that the flow of the control specimen was 115 mm. However, this value was observed to decrease upon the incorporation of SF and RHA into the concrete mixtures. For instance, the mixture incorporating 5% SF and 20% RHA exhibited a flow of 106 mm, and the mixture incorporating 10% SF and 20% RHA exhibited a flow of 104 mm. The higher water requirement of mortar containing SF and RHA for the same flow may be attributed to its porous nature and high surface area as indicated by the density of RHA and SF, which was found to be 2.09 and 2.25, respectively, which is in agreement with the previous studies [47]. Safeer et al. [3] and Christopher et al. [48] also reported a decrease in the mortar flow after incorporating coal bottom ash and biomass ash with cement due to the porous structure of particles and high surface area. Arif et al. [49] also illustrated a decrease in flow with the incorporation of pozzolan materials and required a higher water demand to achieve the targeted workability. C. Medina et al. [50], investigated the impacts of ternary cementitious blends of RHA and FA in concrete. Their research showed that the inclusion of RHA and FA caused an increase in the water demand with an increase in the replacement ratio. This was due to the large surface area of the RHA, resultant of its fineness. Abbas et al. [3] also reported an increase in water demand upon the incorporation of coal ash and it was attributed to its porous nature and irregular particle shape.

Table 6 shows the results of normal consistency and the setting time of mixtures incorporating SF and RHA. The consistency of the control cement paste specimen was 24.03%. It was noticed that the consistency increased with the addition of SF and RHA. For instance, the mixture incorporating 5% SF and 20% RHA showed a consistency of 36.8%, while the mixture incorporating 10% SF and 20% RHA showed a consistency of 37.7%. The higher water demand of silica fume and rice husk ash for the same consistency was due to its hygroscopic nature and high surface area [51,52]. Safeer et al. [41] reported that the normal consistency of cement paste increased from 23% to 37% when 30% RHA was replaced with cement. Similar results were also observed when the normal consistency increased from 27.5 % to 35% for the 20% replacement of SF [52].

The initial and final setting times of the cement were 128 min and 203 min, respectively, as shown in Table 6. It was detected that the initial setting time was reduced with a high replacement level of SF. Incorporating 5% and 10% SF in the mixtures reduced the initial setting time by 43 min and 59 min, respectively. On the further replacement of cement by RHA, the initial setting time increased compared to the mixtures without RHA. A negligible increase in the final setting time was observed when replacing cement with SF and RHA. A similar kind of behavior was also detected in previous research for mixtures incorporating RHA [51,52,53].

### 3.3. Effect of SF and RHA on Compressive and Flexural Strength

**Figure 5:**
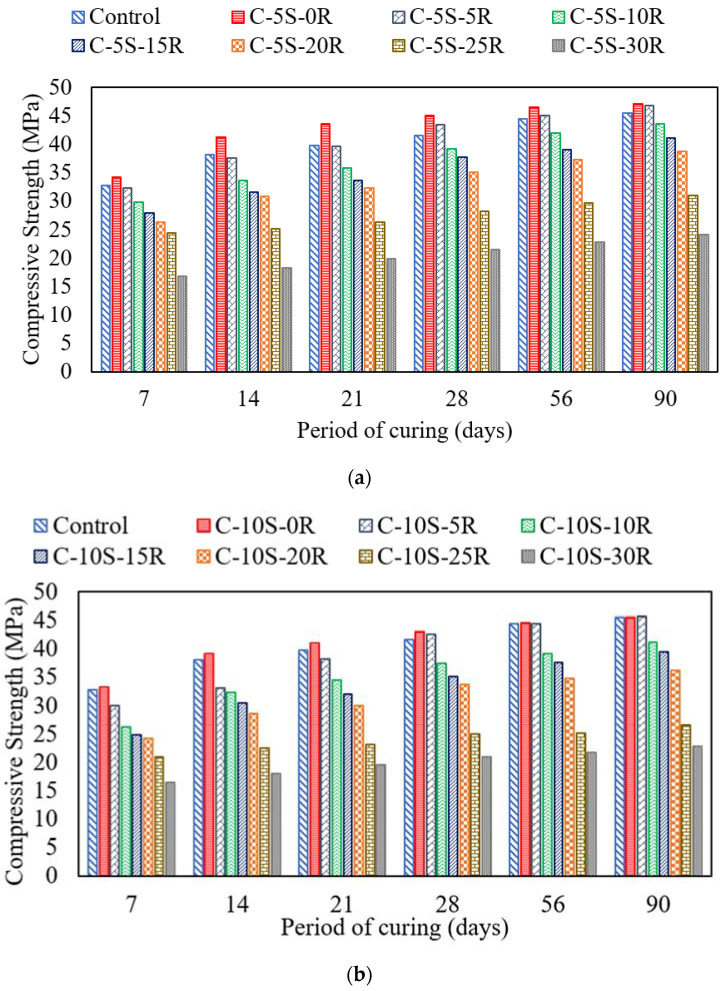
Compressive Strength for specimen incorporating various proportions of SF and RHA. (**a**) C-5S-(0 to 0)R, (**b**) C-10S-(0 to 30)R.

The compressive strength of mixtures incorporating SF and RHA was determined based on five identical specimens. The average compressive strength results (coefficient of variation lesser than 2.7%) of specimens incorporating 5% and 10% SF have been shown in Figure 5a,b, respectively. As expected, an increase in the compressive strength was observed with an increased curing age. For instance, the compressive strength of the control sample increased from 32.7 MPa at 7 days to 41.6 Mpa at 28 days and 45.5 Mpa at 90 days, as shown in Figure 5. Furthermore, an increase in the compressive strength was observed after the addition of SF to the mixture. For instance, at 28 days curing age, on replacing 5% and 10% cement with SF, the compressive strength increased from 41.6 MPa to 45 MPa and 43 MPa, respectively. However, the percentage increase in compressive strength on the addition of 5% SF was around 8% and following the addition of 10%, SF was around 4%. Henceforth, the addition of 5% SF may be preferred as compared to 10% SF. Furthermore, a plateau effect was observed after incorporating RHA into the mixture containing cement and SF. On replacing cement with 5% SF and 5% RHA, the compressive strength was observed to be 43.5 MPa and 46.8 MPa at 28 days and 90 days, respectively. However, after incorporating 5% SF and 10% RHA the compressive strength was observed to be 39.1 MPa and 43.6 MPa at 28 days and 90 days, respectively. A similar decreasing trend was observed in the compressive strength when increasing the RHA content in mixtures containing 5% and 10% SF. Such an increase in the compressive strength caused by the incorporation of SF and RHA may indicate these materials as pozzolanic. The pozzolanic nature of RHA and SF was also mentioned in previous studies [54,55]. Younes et al. [56], produced ternary cementitious blends by incorporating various dosages of rice husk ash along with 20% waste glass powder. A similar plateau effect was reported with the highest compressive strength achieved at 20% waste glass and 5% RHA. The increase in the compressive strength may be attributed to the correct proportion of silica from the RHA and SF with the calcium hydroxide of hydrated cement to make calcium silicate hydrate [16]. Similarly, an increase in compressive strength when incorporating RHA may also be attributed to the filler effect. In agreement with these findings, M.B. Ahsan and Z. Hossain [57] reported that the use of RHA in concrete enhances the workability and strength properties. Replacement of 10% cement with RHA gives better results as compared to higher dosages of RHA. Most of the cube specimen failed in crushing due to the compressive load applied to the specimen.

**Figure 6:**
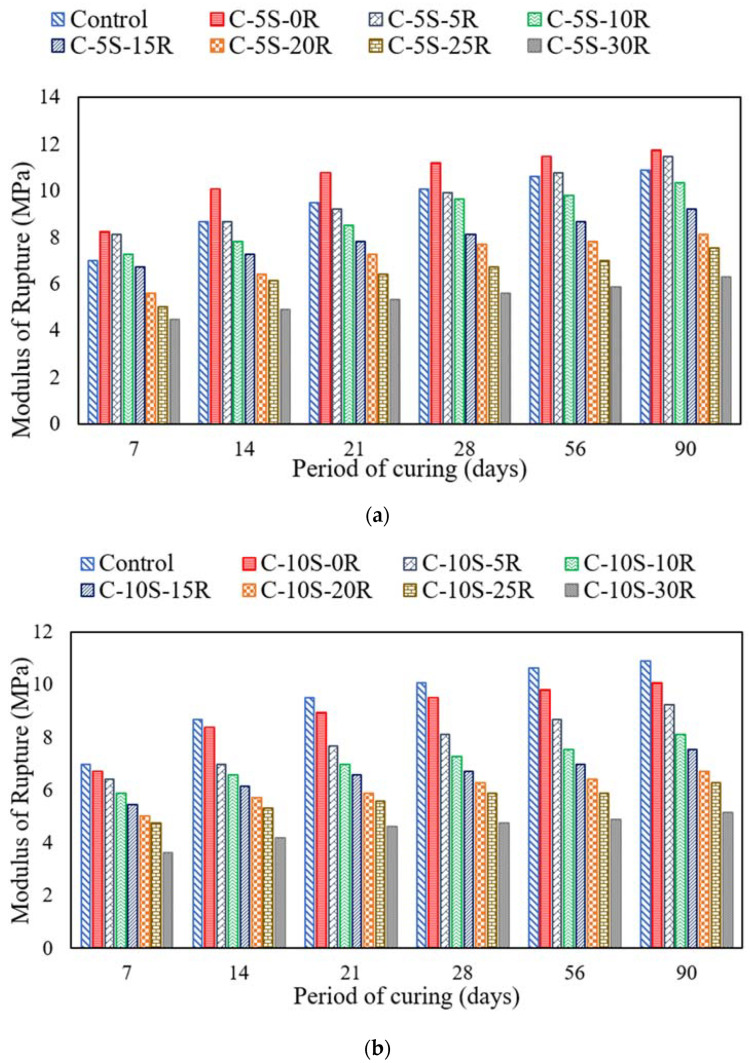
Flexural strength of specimens incorporating various proportions of SF and RHA. (**a**) C-5S-(0 to 30)R, (**b**) C-10S-(0 to 30)R.

Figure 6a,b show the average flexural strength results on five identical specimens for mixtures incorporating various proportions of RHA with 5%SF and 10% SF, respectively. The coefficient of variation was less than 3.91%. A continuous increase in the flexural strength value was observed for mortar prisms with an increase in curing age. For instance, the flexural strength of the control sample was 7 MPa at 7 days, 10 MPa at 28 days, and 10.9 MPa at 90 days. The incorporation of 5% SF along with various proportions of RHA exhibited a plateau effect. After incorporating 5% SF in concrete, the flexural strength increased to 11.2 MPa and 11.76 MPa at 28 days and 90 days, respectively. In addition to a 5% SF replacement, replacing cement with 5% RHA exhibited 9.9 MPa and 11.5 MPa strength at 28 days and 90 days, respectively. Furthermore, on replacing cement with 10% RHA, the flexural strength reduced to 9.7 MPa and 10.4 MPa at 28 days and 90 days, respectively. Thus, a plateau effect was observed similar to the compressive strength case.

After incorporating 10% SF in the concrete mixture, the flexural strength reduced to 9.5 MPa and 10.1 MPa at 28 days and 90 days, respectively. Moreover, incorporating 10% SF along with 10% RHA exhibited a flexural strength of 8.1 MPa and 9.2 MPa at 28 days and 90 days, respectively. The trend of reduction in flexural strength continues until a 30% cement replacement with RHA along with 10% SF replacement. The reduction in flexural strength may be attributed to cement dilution. A similar reduction in flexural strength was reported in previous studies as well. The prime reason reported for such a reduction in flexural strength was cement dilution [3]. The increase in flexural strength of prisms was due to the proper proportion of silica from the RHA and SF with the calcium hydroxide from the cement hydration to improve the microstructure by producing calcium silicate hydrate [58]. The mortar prism specimen demonstrated one prime crack in the centroidal zone.

### 3.4. Expansion due to Alkali-Silica Reaction in Concrete

**Figure 7:**
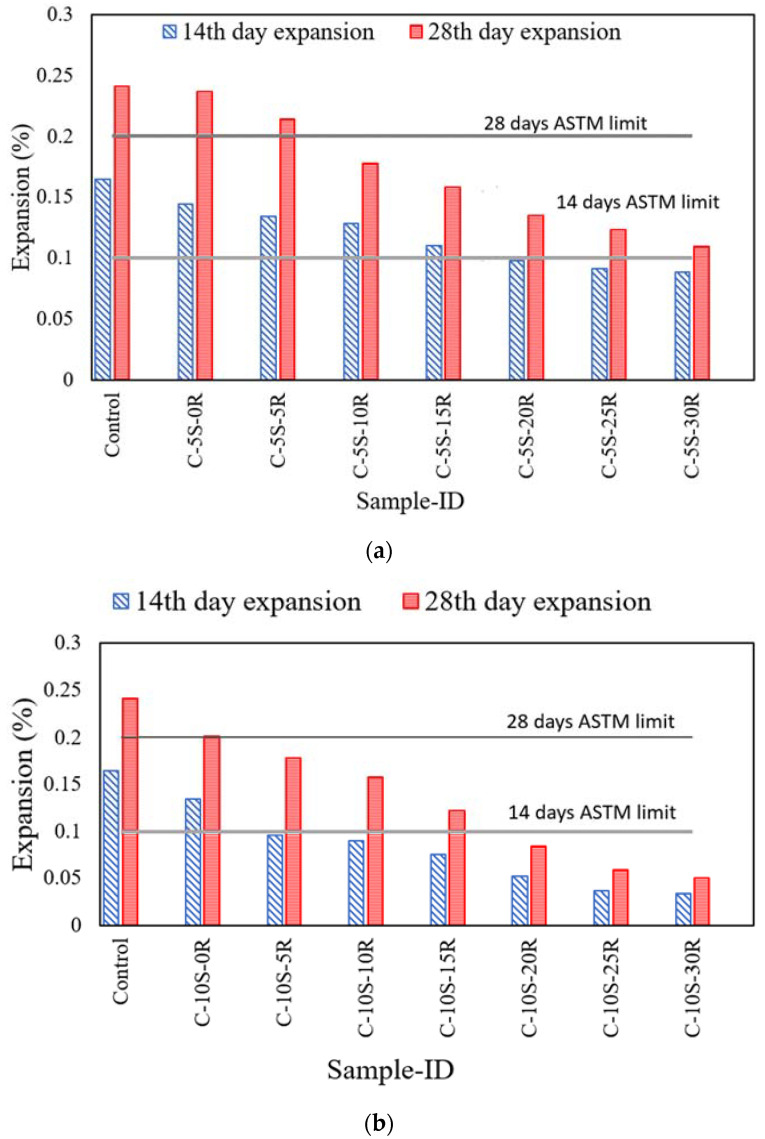
Expansion results of mortar bar incorporating various percentages of SF and RHA. (**a**) C-5S-(0 to 30)R, (**b**) C-10S-(0 to 30).

The results of the accelerated mortar bar test are shown in Figure 7a,b for mixtures incorporating 5% and 10%SF with different percentages of RHA. The average of five samples is presented in the results, with COV of less than 3.8%, which is within the limit of ASTM C1260 [34]. The expansion of the control sample was 0.17% and 0.24% at 14 days and 28 days, respectively. As the expansion exhibited by ternary blends incorporating SF and RHA at various dosages was greater than the limits prescribed by ASTM C 1260, the aggregates can be classified as reactive. The incorporation of SF and RHA resulted in a decrease in expansion. For instance, the specimen incorporating 5% SF and 5% RHA exhibited 0.13% and 0.21% at 14 days and 28 days, respectively. These values are greater than the limits defined by ASTM C 1260. On incorporating 5% SF and 10% RHA an expansion of 0.13% and 0.18% at 14 days and 28 days, respectively, was observed. The 28 days value is less than the limit prescribed by ASTM C 1260. Moreover, the expansion of the mortar bar incorporating 5% SF and 20% RHA was 0.098% and 0.134% at 14 and 28 days, respectively, which was within the limit specified by ASTM C 1260. Similarly, the expansion of the mortar bar incorporating 10% SF and 5% RHA was 0.096 and 0.178% at 14 and 28 days, respectively. This reduction in expansion may be attributed to the high binding power of SF and RHA for alkalis as compared to Portland cement. The pozzolanic behavior of SF and RHA leads to the consumption of portlandite, which in turn supports more negatively charged calcium silicate hydrate which attracts more alkalis. Consequently, the alkali content in the pore solution reduces, causing a further reduction in ASR [13,59]. Afshina and Rangarju [60] reported that the ASR expansion of the mortar bar reduced due to a pozzolanic reaction and the dilution effect of SCMs. A 40% cement replacement with RHA reduced the expansion of the mortar bar by 50% [41]. K. Turk et al. [61] reported that a ternary blend incorporating Fly ash and limestone powder caused a reduction of up to 37% in mortar expansion. 

### 3.5. Effect of ASR on Compressive and Flexural Strengths

**Figure 8:**
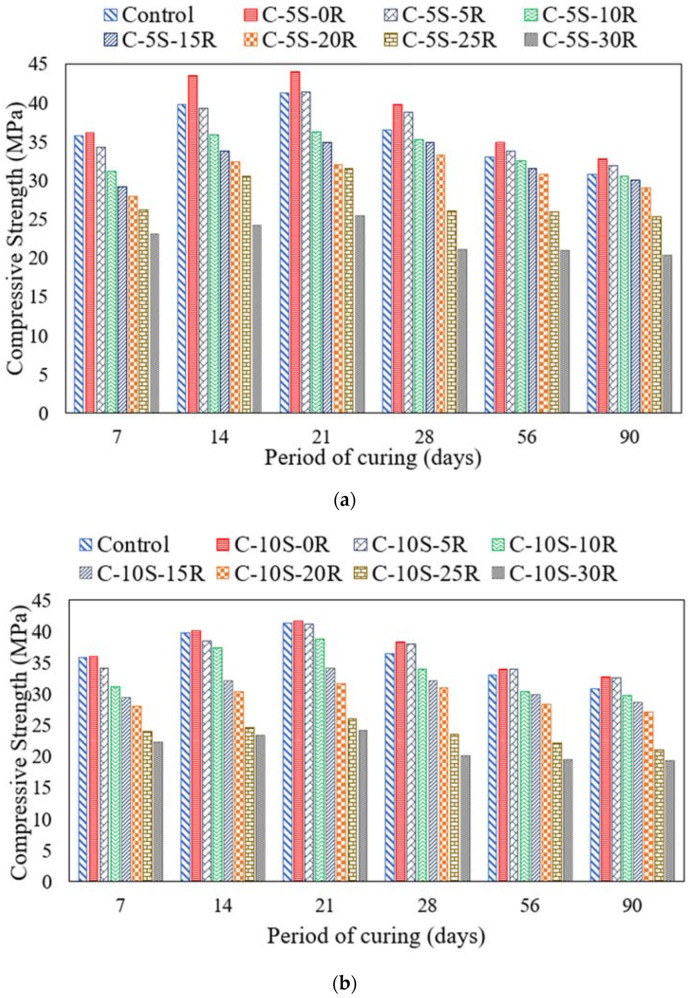
ASR effects on Compressive Strength incorporating various proportions of SF and RHA. (**a**) C-5S-(0 to 30)R, (**b**) C-10S-(0 to 30)R.

Figure 8 shows the impact of ASR on the mechanical properties of concrete. The ASR-prone specimens exposed to ASR conducive conditions (NaOH solution at 80 °C) demonstrated lower compressive strength compared to the identical specimens placed in a normal curing regime. For instance, the control specimen exhibited 36.5 MPa and 30.8 MPa compressive strength at 28 days and 90 days, respectively. The compressive strength of the identical specimens cured under normal regime was 41.6 MPa and 45.5 MPa at 28 days and 90 days, respectively. This accounts for a drop of 12.2% at 28 days and 32.3% at 90 days curing age. Similarly, the specimen incorporating 5% SF and 5% RHA exhibited 31.9 MPa, and the specimen incorporating 5% SF and 10% RHA exhibited 30.5 MPa strength at 90 days. Moreover, the specimen incorporating 10% SF and 5% RHA had 32.6 MPa, and the specimen incorporating 10% SF and 10% RHA showed 29.7 MPa compressive strength. Thus, the compressive strengths of all mixtures were negatively affected by ASR.

**Figure 9:**
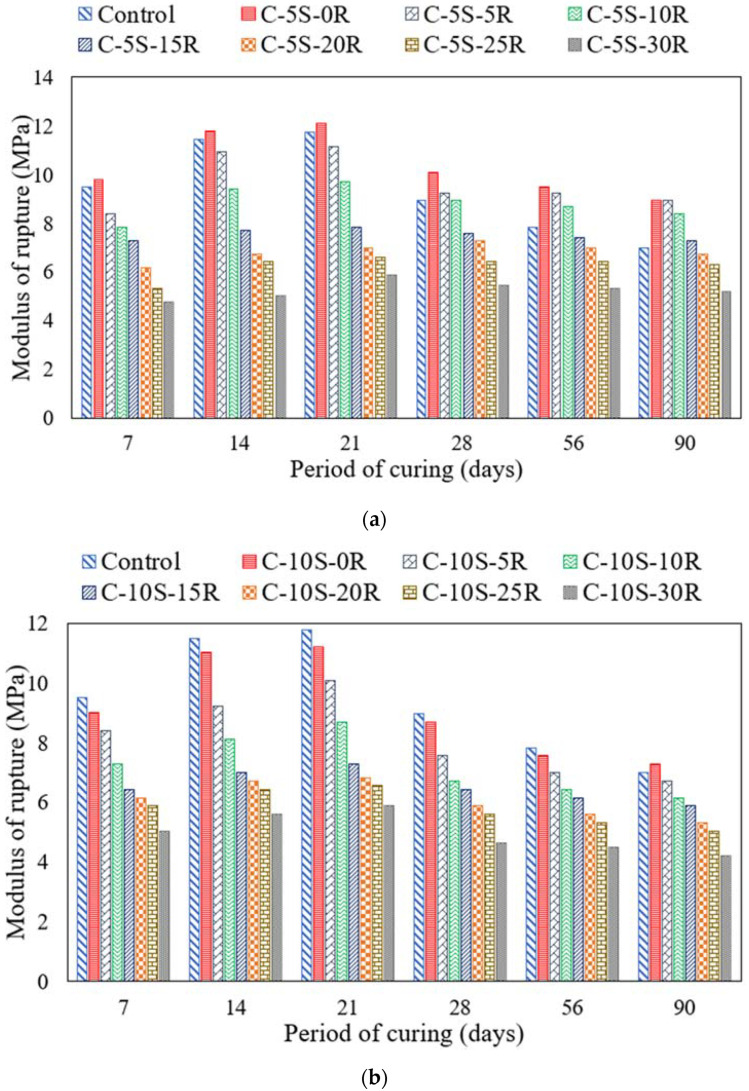
ASR effects on Flexural Strength incorporating various proportions of SF and RHA. (**a**) C-5S-(0 to 30)R, (**b**) C-10S-(0 to 30)R.

Figure 9 shows a similar effect of ASR on the flexural strength of all specimens. For instance, the flexural strength of the control specimen was 8.9 MPa and 7 MPa at 28 days and 90 days, respectively. These values are around 12% and 36% lower than the identical samples cured in a normal water regime. The impact of ASR on the ternary blends was also similar. For instance, the flexural strength of the specimen incorporating 5% SF and 5% RHA was 31.9 MPa, and the flexural strength of the specimen incorporating 10% SF and 5% RHA was 33.9 Mpa, which are less than the values of the identical normal specimen.

It is pertinent to mention that specimens incorporating SF and RHA incurred a lower reduction in compressive and flexural strength as compared to the control specimen. For instance, a specimen incorporating 5% SF along with 20% RHA and a specimen incorporating 10% SF along with 5% RHA subjected to ASR conditions showed a 25% and 28% lesser decrease in compressive strength at 90 days, respectively, as compared to identical specimens cured in normal water. The reduction in mechanical properties may be attributed to the expansion cracks caused by ASR in concrete. A similar reduction in strength owing to ASR conditions has also been reported by previous studies [62,63,64,65]. Saha and Saker [66] reported a reduction in compressive strength for specimens incorporating 20% fly ash subjected to NaOH solution at 80 °C. It predicted that ASR-conducive exposure reduced *f’c* by 20% after a one-year expansion of 0.28% [67].

### 3.6. Thermal Analysis

**Figure 10:**
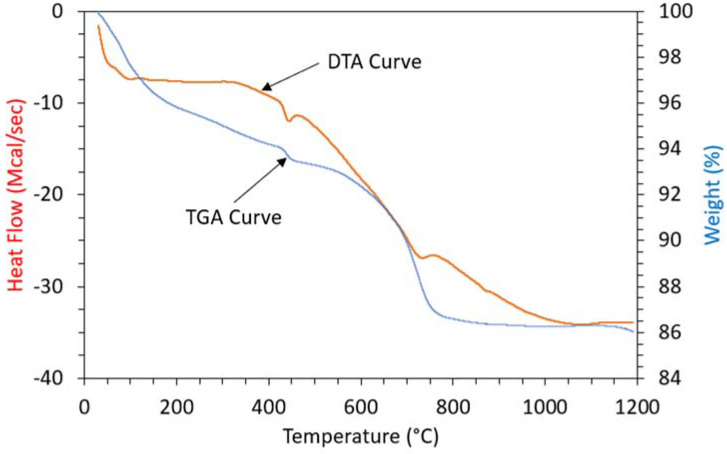
Thermal Analysis of Control Specimen.

Figure 10 shows the thermogravimetric analysis (TGA) and differential thermal analysis (DTA) of the control specimen. An endothermic peak was observed in the DTA curve for the control specimen at around 79 °C due to the removal of hydration water. An endothermic peak at 447 °C indicated the presence of calcium hydroxide (Ca(OH)_2_) in the control sample. Moropoulou et al. [68] also noticed the presence of a (Ca(OH)_2_) peak at around 500 °C. Furthermore, another endothermic peak was noticed at around 740 °C because of the decarbonization reaction. Calcium carbonate (CaCO_3_) decomposition also occurred at around 700 and 900 °C [68].

A mass-loss of 14% in the control specimen indicated by the TGA curve is shown in Figure 10. However, most of the mass-loss was incurred in between 580–800 °C. Mass loss was due to a decrease in the Ca(OH)_2_ content and decarbonation reactions [68].

**Figure 11:**
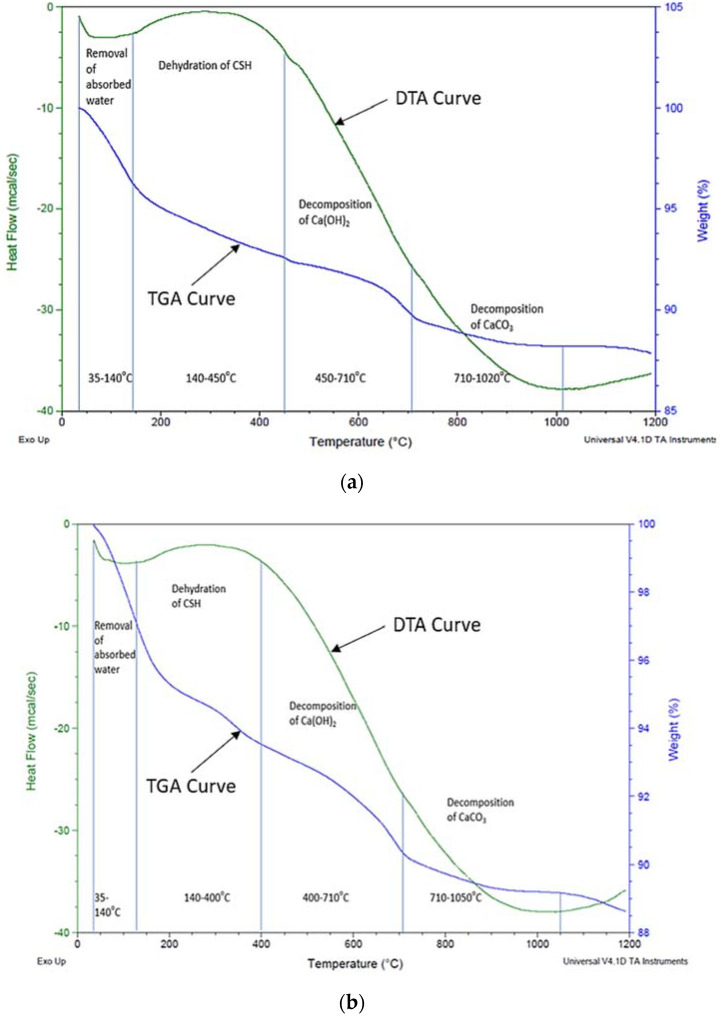
Thermal Analysis of mortar incorporating Silica Fume and Rice Husk Ash. (**a**) C-5S-20R, (**b**) C-10S-5.

Moreover, the DTA curve of specimen C-5S-20R is shown in Figure 11a. An endothermic peak was indicated at around 75 °C, which is related to the evaporation of absorbed water. An exothermic peak was noticed at around 300 °C, which indicated the existence of calcium silicate hydrates [69]. Decomposition of calcium hydro-oxide (Ca(OH)_2_) and calcium carbonate (CaCO_3_) were observed at around 450–710 °C, and 710–1020 °C, respectively. Similarly, the DTA curve of specimen C-10S-5R is shown in Figure 11b. The evaporation of absorbed water around 35–140 °C was observed. An exothermic reaction that occurred at around 140–400 °C is shown in Figure 11b, which can be attributed to the presence of calcium silicate hydrates [69]. The decomposition of calcium hydro-oxide (Ca(OH)_2_) and calcium carbonate (CaCO_3_) was observed at around 400–710 °C, and 710–1050 °C, respectively. Similar results were reported in previous research work [68,70]. TGA curves of C-5S-20R and C-10S-5R specimens are shown in Figure 11. It indicated a total mass loss of 12 and 12.5%, respectively, which is related to the combustion of carbonates.

DTA curves of control and other specimen incorporating SF and RHA was indicated in the exothermic peaks after the replacement of cement with SF and RHA. The presence of calcium silicate hydrate was the reason for these exothermic peaks [41,69]. Moreover, a decrement in mass occurred from 14% to 12% for specimen C-5S-20R and 14% to 12.5% for C-10S-5R relative to the control specimen. This was due to the pozzolanic property of RHA and SF. A reduction in Ca(OH)_2_ occurred because of the pozzolanic behavior of supplementary cementitious materials, as reported by Kandasamy and Shehata [71]. A decrease in Ca(OH)_2_ caused a reduction in the ASR expansion [15,72]. A higher calcium content caused a change in the gel characteristic. Therefore, it enhanced the chances of cracks produced by the ASR [73]. As stated by Kandasamy and Shehata [71], a reduction in Ca(OH)_2_ led to an improvement in the pozzolanic behavior and produced hydration products with a low calcium to silica ratio. The low calcium silica ratio and pozzolanic behavior were attributed to the binding of alkalis, which as result mitigated the expansion produced by the ASR [73]. Afshinnia and Rangaraju [60] noticed a much lower calcium hydroxide in specimens when incorporating 20% of meta-kaolin, due to the improvement of its strength properties. During this research work, a decrement in the amount of Ca(OH)_2_ was also noticed for C-5S-20R and C-10S-5R.

## 4. Conclusions

This study provides an attempt to explore the possible utilization of waste materials in the form of ternary blends to control the negative impact of ASR on the mechanical properties of concrete. Various proportions of SF and RHA were incorporated into concrete as cement replacements to examine their effectiveness in controlling ASR. Based on the results of this research, the following conclusions were drawn:

- The incorporation of waste ashes (SF and RHA) reduces the flow of concrete mixtures due to the porous nature and increased surface area.
- The incorporation of 5% or 10% SF along with 5% RHA exhibited improved compressive strength at 28 days of curing age. However, after increasing the waste ash content, the compressive strength was reduced. Similarly, this behavior was also observed in the case of flexural strength.
- A decrease in the expansion of the mortar bar was noticed with an increase in the SF and RHA replacement due to pozzolanic reaction and dilution effect. The expansion of the mortar bar incorporating 5% SF and 20% RHA was 0.10% and 0.13% at 14 days and 28 days, respectively, which were less than the limit specified by ASTM C 1260. Moreover, the expansion of the mortar bar when incorporating 10% SF and 5% RHA was 0.096 and 0.178% at 14 and 28 days, respectively.

In short, it is concluded that a ternary blend of SF along with RHA may be considered as a practical approach to mitigate ASR with the lowest level of compromise in terms of mechanical properties. Although a few proportions have been reported to be effective, further study can be carried out to determine proportions corresponding to the available materials for desired projects.

## 5. Future Recommendations

- A durability test should be conducted and the sulfate attack and chloride penetration of concrete incorporating SF and RHA should be studied.
- A more detailed study on effect of SF and RHA on the alkalinity of mortar may be studied by alkali leaching.
- The effects of using ternary blends of other pozzolanic materials should be studied.

## Figures and Tables

**Table 1:** Mixture Proportion used to prepare sample.

Sample	Cement	Silica Fume	Rice Husk Ash
	%	%	%
Control	100	0	0
C-5S-0R	95	5	0
C-5S-5R	90	5	5
C-5S-10R	85	5	10
C-5S-15R	80	5	15
C-5S-20R	75	5	20
C-5S-25R	70	5	25
C-5S-30R	65	5	30
C-10S-0R	90	10	0
C-10S-5R	85	10	5
C-10S-10R	80	10	10
C-10S-15R	75	10	15
C-10S-20R	70	10	20
C-10S-25R	65	10	25
C-10S-30R	60	10	30

**Table 2:** Chemical composition of cement, SF, and RHA.

Oxides	Cement	SF	RHA
CaO	59.62	1.23	0.65
MgO	3.85	0.89	1.43
SiO_2_	21.73	90.13	88.51
Al_2_O_3_	4.87	0.42	0.21
Fe_2_O_3_	2.12	0.515	0.61
SO_3_	1.65	1.23	0.40
K_2_O	0.67	1.51	1.89
Na_2_O	0.21	0.512	0.18
L. O. I	2.61	2.08	2.89

**Table 3:** Physical Properties of Cement, Silica Fume, and Rice Husk Ash.

Property	Standard	Cement	SF	RHA
Specific Gravity	ASTM C188	3.12	2.25	2.09
Unit Weight (Kg/m^3^)	ASTM C29	1428	580.4	554.8
Fineness (passing No. 200)	ASTM C184	96.5%	100%	100%
Autoclave expansion	ASTM C151	0.11%	-	-

**Table 4:** Physical Properties of Aggregates.

Property	Standard	Value
Impact Value	BS-812	22%
Crushing Value	BS-812	26%
Abrasion Test	ASTM C 535	28%
Specific Gravity	ASTM C 127	2.58
Water Absorption (%)	ASTM C 127	0.96
Bulk Density (Kg/m^3^)	ASTM C 29	1393
Voids Content (%)	ASTM C 29	36.75%

**Table 5:** Results of Flowability of mortar incorporating SF and RHA.

Sr.	Sample	Cement	SF	RHA	Aggregates	Water	Flow Dia
No	-	(Grams)	(Grams)	(Grams)	(Grams)	(Grams)	(mm)
1	C	220	0	0	495	103.4	115
2	C-5S-0R	209	11	0	495	103.4	114
3	C-5S-5R	198	11	11	495	103.4	113
4	C-5S-10R	187	11	22	495	103.4	112
5	C-5S-15R	176	11	33	495	103.4	109
6	C-5S-20R	165	11	44	495	103.4	106
7	C-5S-25R	154	11	55	495	103.4	104
8	C-5S-30R	143	11	66	495	103.4	102
9	C-10S-0R	198	22	0	495	103.4	113
10	C-10S-5R	187	22	11	495	103.4	112
11	C-10S-10R	176	22	22	495	103.4	111
12	C-10S-15R	165	22	33	495	103.4	107
13	C-10S-20R	154	22	44	495	103.4	104
14	C-10S-25R	143	22	55	495	103.4	103
15	C-10S-30R	132	22	66	495	103.4	101

**Table 6:** Fresh Properties of Cement.

Sr. No	Sample	Normal Consistency	Initial Setting Time	Final Setting Time
-	-	(%)	(Minutes)	(Minutes)
1	C	24.03	128	203
2	C-5S-0R	28.8	85	215
3	C-5S-10R	32.7	104	228
4	C-5S-20R	36.8	124	243
5	C-5S-30R	40.7	147	265
6	C-10S-0R	30.3	69	211
7	C-10S-10R	33.4	90	225
8	C-10S-20R	37.7	108	239
9	C-10S-30R	43	132	258

## Data Availability

Not applicable.
